# Geographic Distribution and Genetic Characterization of Lassa Virus in Sub-Saharan Mali

**DOI:** 10.1371/journal.pntd.0002582

**Published:** 2013-12-05

**Authors:** David Safronetz, Nafomon Sogoba, Job E. Lopez, Ousmane Maiga, Eric Dahlstrom, Marko Zivcec, Friederike Feldmann, Elaine Haddock, Robert J. Fischer, Jennifer M. Anderson, Vincent J. Munster, Luis Branco, Robert Garry, Stephen F. Porcella, Tom G. Schwan, Heinz Feldmann

**Affiliations:** 1 Laboratory of Virology, Rocky Mountain Laboratories, National Institute of Allergy and Infectious Diseases, National Institutes of Health, Hamilton, Montana, United States of America; 2 Malaria Research and Training Center, University of Sciences, Techniques and Technologies of Bamako, Bamako, Mali; 3 Laboratory of Zoonotic Pathogens, Rocky Mountain Laboratories, National Institute of Allergy and Infectious Diseases, National Institutes of Health, Hamilton, Montana, United States of America; 4 Rocky Mountain Laboratory Research Technologies Section, Genomics Unit, Rocky Mountain Laboratories, National Institute of Allergy and Infectious Diseases, National Institutes of Health, Hamilton, Montana, United States of America; 5 Department of Medical Microbiology, University of Manitoba, Winnipeg, Manitoba, Canada; 6 Rocky Mountain Veterinary Branch, Rocky Mountain Laboratories, National Institute of Allergy and Infectious Diseases, National Institutes of Health, Hamilton, Montana, United States of America; 7 Laboratory of Malaria and Vector Research, National Institute of Allergy and Infectious Diseases, National Institutes of Health, Rockville, Maryland, United States of America; 8 Department of Microbiology and Immunology, Tulane School of Medicine, New Orleans, Louisiana, United States of America; 9 Autoimmune Technologies LLC, New Orleans, Louisiana, United States of America; Centers for Disease Control and Prevention, United States of America

## Abstract

**Background:**

Lassa fever is an acute viral illness characterized by multi-organ failure and hemorrhagic manifestations. Lassa fever is most frequently diagnosed in Nigeria, Sierra Leone, Liberia, and Guinea, although sporadic cases have been recorded in other West African countries, including Mali. The etiological agent of Lassa fever is Lassa virus (LASV), an *Arenavirus* which is maintained in nature and frequently transmitted to humans by *Mastomys natalensis*. The purpose of this study was to better define the geographic distribution of LASV-infected rodents in sub-Saharan Mali.

**Methodologies/Principal Findings:**

Small mammals were live-trapped at various locations across Mali for the purpose of identifying potential zoonotic pathogens. Serological and molecular assays were employed and determined LASV infected rodents were exclusively found in the southern Mali near the border of Côte d'Ivoire. Overall, 19.4% of *Mastomys natalensis* sampled in this region had evidence of LASV infection, with prevalence rates for individual villages ranging from 0 to 52%. Full-length genomic sequences were determined using high throughput sequencing methodologies for LASV isolates generated from tissue samples of rodents collected in four villages and confirmed the phylogenetic clustering of Malian LASV with strain AV.

**Conclusions/Significance:**

The risk of human infections with LASV is greatest in villages in southern Mali. Lassa fever should be considered in the differential diagnosis for febrile individuals and appropriate diagnostic techniques need to be established to determine the incidence of infection and disease in these regions.

## Author Summary

Lassa fever is an acute infection associated with hemorrhagic manifestations and multi-organ failure in West Africa. The etiological agent of Lassa fever is Lassa virus (LASV), a rodent-borne arenavirus, which is maintained in nature and transmitted to humans by the multimammate rat, *Mastomys natalensis*. Despite the ubiquitous nature of the rodent reservoir, LASV-infected animals are most commonly documented in Nigeria, Sierra Leone, Guinea and Liberia. These four countries represent the historic endemic region for Lassa fever, although there is increasing evidence of sporadic cases occurring in other West African nations including Mali. To better define the geographic distribution of LASV-infected rodents in Mali, we tested samples from small animals collected at 27 sites across the country. Although *M. natalensis* was the predominant rodent species in the majority of villages, evidence of LASV infection was exclusively found in southern Mali, where overall nearly 20% of rodents were positive. The full genomic sequence was determined for five isolates and confirmed LASV in Mali is closely related to strain AV. We conclude that there is a risk of human exposure to LASV in villages in southern Mali and Lassa fever should be considered in the differential diagnosis for acutely ill, febrile patients.

## Introduction

Lassa fever is an acute viral illness that is associated with a wide range of disease manifestations. While the majority of human cases are asymptomatic or mild in nature, approximately 20% of infections demonstrate moderate to severe symptoms, which can include acute hemorrhagic fever characterized by multi-organ failure [1]. Lassa fever has an incidence ranging from 300,000 to 500,000 cases per annum with approximately 5000 deaths [2].

The etiological agent of Lassa fever is Lassa virus (LASV), a rodent-borne pathogen belonging to the *Arenavirus* genus within the *Arenaviridae* [3], [4]. The natural reservoir of LASV is the multimammate rat (*Mastomys natalensis*), which during infection with LASV sheds copious amounts of virus in urine [1], [5]. Humans primarily become infected with LASV following inhalation or ingestion of virus-contaminated materials, though person-to-person transmission is also well documented, especially in nosocomial settings where mortality rates are often increased [5], [6]. Although *M. natalensis* are ubiquitous in many parts of sub-Saharan Africa, LASV-infected rodents appear to be restricted to West African countries, notably Nigeria, Sierra Leone, Liberia, and Guinea [5], [7]–[9]. To date, outbreaks have been confined to the endemic region consisting of these four countries, though evidence of LASV infections and sporadic cases of Lassa fever have been reported from other West and Central African countries [10]–[14]. Additionally, LASV has been introduced into Europe and North America several times over the past four decades, making Lassa fever one of the most prominent imported exotic viral hemorrhagic fevers with a high impact on national public health [12]. In this regard, LASV strain AV was isolated from a fatal case returning home from travel through Côte d'Ivoire, Burkina Faso, and Ghana [15]. More recently, another imported Lassa fever case was diagnosed post-mortem, in a young man with a 10-day history of fever who was medically evacuated from Mali to London [16]. Subsequent field studies conducted in the village of Soromba, where the man was living and working in Mali, demonstrated the presence of LASV-infected rodents [17]. The purpose of this study was to expand upon these original findings and better define the geographical distribution of LASV-infected *M. natalensis* in sub-Saharan Mali as well as provide an in-depth genetic characterization of novel LASV isolates from this region.

## Methods

### Ethics statement

This research was carried out in accordance with protocols approved by an Institutional Animal Care and Use Committee of the National Institutes of Health (study protocol #'s 2008-1, 2010-78 and 2011-48). Animal work was conducted adhering to the institution's guidelines for animal use, and followed the guidelines and basic principles in the United States Public health Service Policy on Humane Care and Use of Laboratory Animals, and the Guide for the Care and Use of Laboratory Animals. Residents in the villages gave informed consent prior to our setting traps in their houses.

### Biosafety

Personal protective equipment (PPE) utilized in these studies was in accordance with established institutional guidelines to prevent exposure to rodent-borne pathogens. In areas of known or suspected LASV circulation, additional PPE was utilized including double gloves, Tyvek coveralls and gowns and HEPA filtered personal powered air purifying respirators with full head covers. Each day at the conclusion of sample processing, traps were disinfected in a mild bleach solution for a minimum of 10 minutes and triple-rinsed with clean water. The work station and all equipment were similarly disinfected. Animal carcasses were incinerated on site.

### Sample collection and processing

**Figure 1:**
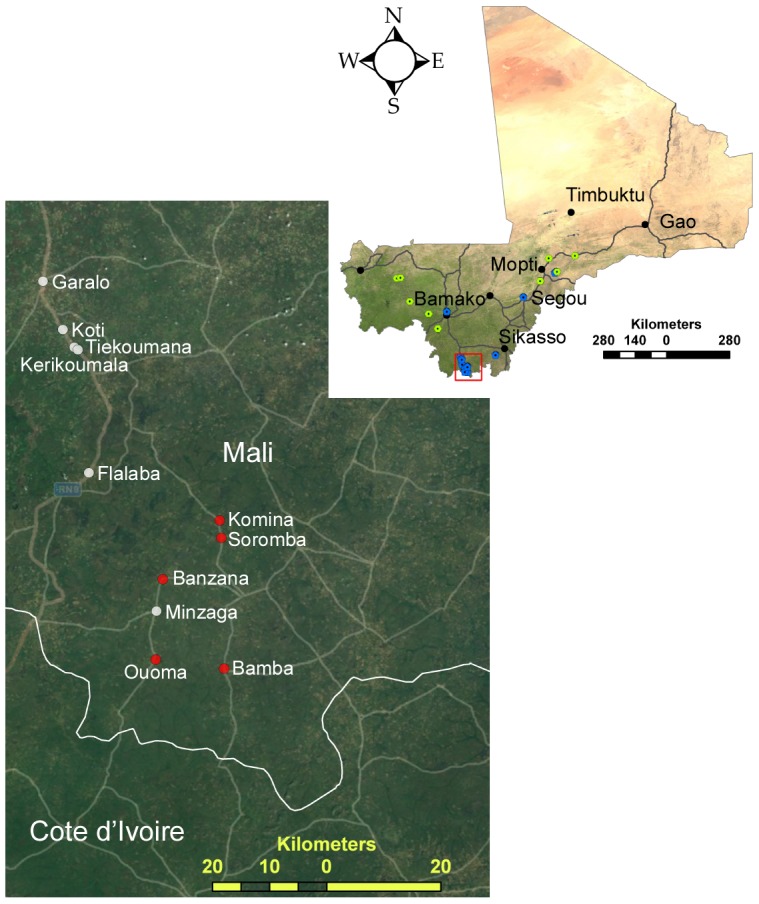
Map of Mali depicting the various locations where small mammals were trapped. Sera and tissue samples were collected at some locations (blue dots) whereas at other locations only sera were collected (green dots). The major cities of Mali are represented by black markers. Inset is an expanded view of southern Mali where the occurrence of Lassa virus infected rodents was documented. The sampled villages are named, and red markers indicate villages where Lassa virus infected rodents were documented. The maps were generated using ESRI ArcMap 10.1; the map of Mali utilizes an ESRI satellite imagery basemap and the inset utilizes an ESRI basemap with the imagery and transportation layers activated, both supplied with the GIS software.

Between December 2007 and March 2012, six field expeditions were conducted to capture and sample small mammals in peridomestic settings from across Mali for the purposes of testing them for zoonotic pathogens [17], [18](Figure 1, Table 1). Trapping was conducted in various ecozones of sub-Saharan Mali including the grasslands and croplands south of the Sahel to the open and wooded savannas of Southern Mali [19]. Small mammals were live-trapped at each location for one to three consecutive nights using Sherman traps (H.B. Sherman Traps) baited with a mixture of onions and roasted peanuts and set in areas with known or suspected rodent activity. The following morning traps were collected and captured animals immediately processed onsite. Small mammals were anaesthetized by inhalation of isoflurane and exsanguinated via cardiac puncture. The weight, sex and relative age (inferred from body weight and developmental stage) were recorded for each rodent and an ear punch collected and stored in 70% ethanol for cytochrome B sequencing.

**Table 1:** Prevalence of *Mastomys natalensis* captured in various villages in sub-Saharan Mali.

Site	Latitude	Longitude	Trapping dates	Total # trap nights	Total # captures (# species)	# *M. natalensis* (% total)
Petaka	15°01′25″N	02°50′55″W	18 Jan 09	73	39 (8)	6 (15.4)
Sama	14°55′25″N	03°53′50″W	7 Dec 07	58	6 (3)	0 (0)
Sinkerma	14°22′51″N	03°34′06″W	17 Jan 09	75	16 (6)	3 (18.8)
Sefeto West	14°08′26″N	09°49′37″W	8 Dec 07	78	3 (3)	0 (0)
Djougounte	14°07′19″N	09°58′33″W	9 Dec 07	78	9 (4)	0 (0)
Senosa	14°00′24″N	04°14′34″W	5 Dec 07	58	2 (1)	0 (0)
Molibana	14°00′56″N	04°13′52″W	6 Dec 07	58	24 (4)	1 (4.2)
Doukombou	14°21′19″N	03°39′26″W	30 Sept 11	80	40 (2)	35 (87.5)
Doukombou	14°21′19″N	03°39′26″W	2 Oct 11	80	11 (1)	11 (100)
Kalibombo	14°24′01″N	03°36′02″W	1 Oct 11	100	35 (2)	33 (94.2)
Kerikoumala	10°53′22″N	07°23′16″W	5 Oct 11	84	26 (1)	26 (100)
Djidian	13°12′03″N	09°27′14″W	11 Dec 07	78	19 (4)	10 (52.6)
Belenikegny	13°22′57″N	04°55′00″W	19 Jan 09	73	27 (5)	5 (18.5)
Belenikegny	13°22′57″N	04°55′00″W	5–6 Jan 10	200	111 (7)	13 (11.7)
Doneguebougou	12°48′18″N	07°58′49″W	12–14 June 09	251	53 (7)	38 (71.7)
Doneguebougou	12°48′18″N	07°58′49″W	25–26 Sept 11	160	41 (1)	41 (100)
Bozokin	12°41′53″N	08°41′53″W	12 Jan 09	74	19 (1)	19 (100)
Kenieroba	12°06′44″N	08°19′56″W	13 Jan 09	71	21 (4)	8 (38.1)
Fourda	12°05′29″N	08°20′06″W	14 Jan 09	74	17 (2)	14 (82.4)
N'Tessoni	11°04′19″N	06°01′37″W	5–6 June 09	168	25 (6)	15 (60)
Garalo	10°59′37″N	07°26′14″W	5–6 Oct 11	182	22 (1)	22 (100)
Kotié	10°55′03″N	07°24′21″W	6 Oct 11	76	11 (1)	11 (100)
Tiekoumana	10°53′08″N	07°22′56″W	2 Mar 12	120	12 (2)	11 (91.7)
Soromba	10°35′21″N	07°09′21″W	8–9 June 09	167	25 (1)	25 (100)
Soromba	10°35′21″N	07°09′21″W	8–9 Jan 10	120	21 (1)	21 (100)
Soromba	10°35′21″N	07°09′21″W	3 Mar 12	109	13 (1)	13 (100)
Komina	10°36′59″N	07°09′30″W	8–9 Jan 10	80	12 (1)	12 (100)
Komina	10°36′59″N	07°09′30″W	3 Mar 12	74	7 (1)	7 (100)
Minzaga	10°31′26″N	07°14′53″W	4–5 Mar 12	160	12 (1)	12 (100)
Banzana	10°31′26″N	07°14′53″W	4–5 Mar 12	210	28 (1)	28 (100)
Bamba	10°22′59″N	07°09′06″W	6–7 Mar 12	198	36 (2)	35 (97.2)
Ouoma	10°23′50″N	07°15′33″W	6–7 Mar 12	180	16 (1)	16 (100)
Flalaba	10°41′30″N	07°21′53″W	8 Mar 12	160	34 (1)	34 (100)

At some locations (Figure 1) necropsies were performed on animals and liver samples collected for virus detection. Tissue samples were divided into two pieces; one was immediately frozen and stored in liquid nitrogen (for virus isolation), while the other half was submerged in 600 µl of buffer RLT (Qiagen), mechanically homogenized using a bullet blender (Next Advance) and frozen in liquid nitrogen. Immediately after collection, 140 µl of whole blood was inactivated with lysis buffer AVL (Qiagen) and subsequently frozen. The remaining blood was allowed to clot, spun-down and sera removed while in the field. At the conclusion of the field work, samples were stored at −80°C and shipped on liquid nitrogen to the BSL-4 facility at the Rocky Mountain Laboratories (RML) of the Division of Intramural Research, National Institute of Allergy and Infectious Diseases, NIH.

### Rodent speciation

Genomic DNA was extracted from 3 mm ear punches using DNeasy kits (Qiagen) and speciation of rodents was genetically confirmed by cytochrome B sequence analysis using primers L14723 and H15915, essentially as previously described [18], [20].

### Serology

Serum samples were tested for the presence of anti-LASV IgG antibodies using an enzyme linked immunosorbent assay (ELISA) based on a recombinant nucleocapsid protein antigen derived from LASV Josiah [21] and using a mixture of anti-rat and anti-*Peromyscus leucopus* secondary antibodies (each at 1∶2,000 dilution, KPL). Samples were initially screened at a 1∶100 dilution and were considered seropositive if they yielded an OD_405_ value greater than three times the standard deviation of seronegative controls. Reactive samples were titered using 4-fold dilutions.

### RT-PCR

Total RNA was extracted from inactivated tissue and blood samples using RNeasy and QIAamp viral RNA kits (Qiagen), respectively. These extractions were screened for the presence of LASV RNA using real-time and conventional RT-PCR assays as previously described [17], [22].

### Virus isolation

Tissue homogenates were prepared from selected seronegative rodents which had detectable LASV RNA in liver and blood samples and passaged twice in Vero E6 cells. For the initial passage, homogenates were diluted 1∶2,500 and incubated on cells for 4 days. Supernatant from p1 was diluted 1∶1,000 and passaged a second time (p2) on nearly confluent monolayers of Vero E6 cells. Cells were monitored daily for CPE and supernatant was harvested on day 5 post-infection. LASV isolation was confirmed by RT-PCR as outlined above.

### High throughput sequencing

The full-length genomic sequence of five LASV isolates from Mali, including the original isolate (Soromba-R) obtained in 2009 [17], was determined using high throughput sequencing technologies. Approximately 28 mL of p2 supernatant of each isolate was centrifuged at 25,000×G for 2 hrs in a SW-28 rotor. The resulting viral pellets were lysed in buffer RLT containing 145 mM β-mercaptoethanol and RNA was extracted and purified using RNeasy mini-columns according to the manufacturer's protocol (Qiagen). cDNA was synthesized and libraries generated and quantified as previously described [23]. The cDNA libraries were normalized to 1×10^7^molecules/µL and pooled. Preparation of templated beads for 454 sequencing followed the emPCR Method Manual-Lib-L-LV (Roche Applied Science). Library pools were added to DNA Capture Beads at a target of 1.0 copy per bead. Enriched DNA Capture Beads were then sequenced on a 454 Titanium instrument (Roche Applied Science) per the manufacturer's instructions using a 2-region gasket.

Genomic viral sequences on the Genome Sequencer FLX generated 18,000 usable fragment reads with 275-fold coverage. De Novo genome assembly was performed using GS De Novo Assembler v2.6 (454 Life Sciences) and CLC Genomics Workbench 4.0 (CLC Bio). Translated BLAST (blastx) was performed to eliminate non-viral contaminants and assembly was performed using Sequencher v5.0 (Gene Codes). Assembled contigs were refined by mapping the 454 reads using GS Reference Mapper v2.6 (454 Life Sciences). Full length sequences of the nucleocapsid protein, glycoproteins and polymerase genes were aligned using Clustal 2.1 multiple sequence alignment program (Conway Institute UCD) with the ClustalW algorithm and phylogenetic trees were constructed using Geneious Tree builder v6.51 (Biomatters Ltd.) with the Jukes-Cantor Neighbor-joining method with bootstrapping to 10,000 iterations.

## Results

### Rodent diversity

Between December 2007 and March 2012, a total of 793 small animals were captured in sub-Saharan Mali for the purpose of screening for zoonotic pathogens. As previously described, 14 different species of rodents and shrews were captured in Mali [18]. *Mastomys natalensis* was the most frequently captured small mammal at the majority of locations (Table 1).The diversity of captures was greatest in the northern regions, especially around the Niger inland delta. In contrast, little or no rodent diversity was observed in the southern regions of Mali around the village of Soromba where, during three field expeditions, all but two animals captured in these areas were *M. natalensis* (246/248, 99.2%). Representative cytochrome b sequences (deposited to GenBank) as well as skull voucher specimens (deposited to the Smithsonian Institution) for these animals are described elsewhere [17], [18].

### Detection of LASV antibodies and RNA in Malian rodents

Serum samples from 715 rodents, including 511 from *M. natalensis*, were tested for the presence of antibodies reactive to a recombinant LASV nucleocapsid antigen by standard ELISA methodologies. Shrew samples were not evaluated due to a lack of specificity of the secondary antibodies and sample volumes from some (mostly juvenile) rodents were of insufficient quantity to perform serological analysis. Overall, 203 of the 204 serum samples tested from rodents other than *M. natalensis* were seronegative. The lone sample that was reactive had a serological titer of 1600 and was collected from an adult male African grass rat (*Arvicanthis niloticus*) captured in Belenikegny, a small fishing village on the Bani River (Figure 1). Although we were unable to determine the infecting arenavirus, it was most likely Ippy virus which is antigenically related to LASV and is maintained in nature in *Arvicanthis* species [24], [25]. Of the 511 serum samples from *M. natalensis*, a total of 35 were positive for an overall seroprevalence of 6.8%. Geographically, serological evidence of LASV infection was limited to southern Mali, specifically the villages near Soromba, where the overall prevalence increased to 14.2% (35 of 246). The seroprevalence rates for individual villages in this region varied from 0 to 48% (Table 2).

**Table 2:** Summary of serological and molecular testing for Lassa virus conducted on *Mastomys natalensis* captured in southern Mali.

Village	Dates	Serological results # pos./# tested (%)	RT-PCR results # pos./# tested (%)	Combined # of *M. natalensis* with evidence of LASV infection (%)
Garalo	5–6 Oct 11	0/22	0/22	0
Kotié	6 Oct 11	0/11	0/11	0
Tiekoumana	2 Mar 12	0/11	0/11	0
Soromba	8–9 June 09	12/25 (48)	6/25 (24)*	13/25 (52)
Soromba	8–9 Jan 10	6/21 (28.6)	2/21 (9.5)	7/21 (33.3)
Soromba	3 Mar 12	2/13 (15.4)	2/13 (15.4)	4/13 (30.7)
Komina	8–9 Jan 10	3/12 (25)	0/12	3/12 (25)
Komina	3 Mar 12	0/7	1/7 (14.3)	1/7 (14.3)
Minzaga	4–5 Mar 12	0/12	0/12	0
Banzana	4–5 Mar 12	1/28 (3.6)	0/28	1/28 (3.6)
Bamba	6–7 Mar 12	6/35 (17.1)	7/35 (20)	13/35 (37.1)
Ouoma	6–7 Mar 12	5/16 (31.3)	1/16 (6.3)	6/16 (37.5)
Flalaba	8 Mar 12	0/33	0/34	0
Total		35/246 (14.2)	19/248 (7.7)	48/248 (19.4)

* As originally reported [17].

Tissue samples were collected from 591 rodents and tested for the presence of LASV RNA by real-time and conventional RT-PCR assays. Similar to the serological results, molecular evidence of LASV infection was only found in rodents collected in southern Mali with a total of 19 positive animals identified for an overall prevalence of 7.7% (19 of 246). Prevalence rates for individual villages ranged from 0 to 24% (Table 2). Of these 19 infected animals, 6 were also seropositive. Interestingly, the majority of seropositive animals (29 of 35, 82.9%) did not have detectable viral RNA in blood or liver samples (Table 2).

Combining the results of serological and molecular testing, a total of 48 (19.4%) rodents, all *M. natalensis*, demonstrated evidence of LASV infection. Twenty-nine animals were seropositive and RT-PCR negative; six animals were both seropositive and RT-PCR positive; and the remaining thirteen animals were seronegative but RT-PCR positive (Table 2). The majority of these animals were adult (41 of 48, 85.4%), with roughly an equal distribution of males and females (22 of 48, 45.8% compared with 26 of 48, 54.2%, respectively) (Table 3).

**Table 3:** Characteristics of *Mastomys natalensis* with evidence of Lassa virus infection.

Infection status		Total	Sex No. (%)		Age No. (%)		Serological titers
Serology	RT-PCR		Male	Female	Adult	Sub-adult	
Neg.	Pos.	13	5 (38.5)	8 (61.5)	10 (76.9)	3 (23.1)	<100 (n = 13)
Pos.	Pos.	6	3 (50)	3 (50)	5 (83.3)	1 (16.7)	100 (n = 3), 400 (n = 2), 1600 (n = 1)
Pos.	Neg.	29	14 (48.3)	15 (51.7)	26 (89.7)	3 (10.3)	100 (n = 4), 400 (n = 10), 1600 (n = 7), ≥6400 (n = 8)

### Isolation and genetic analysis of LASV from Malian rodents

**Figure 2:**
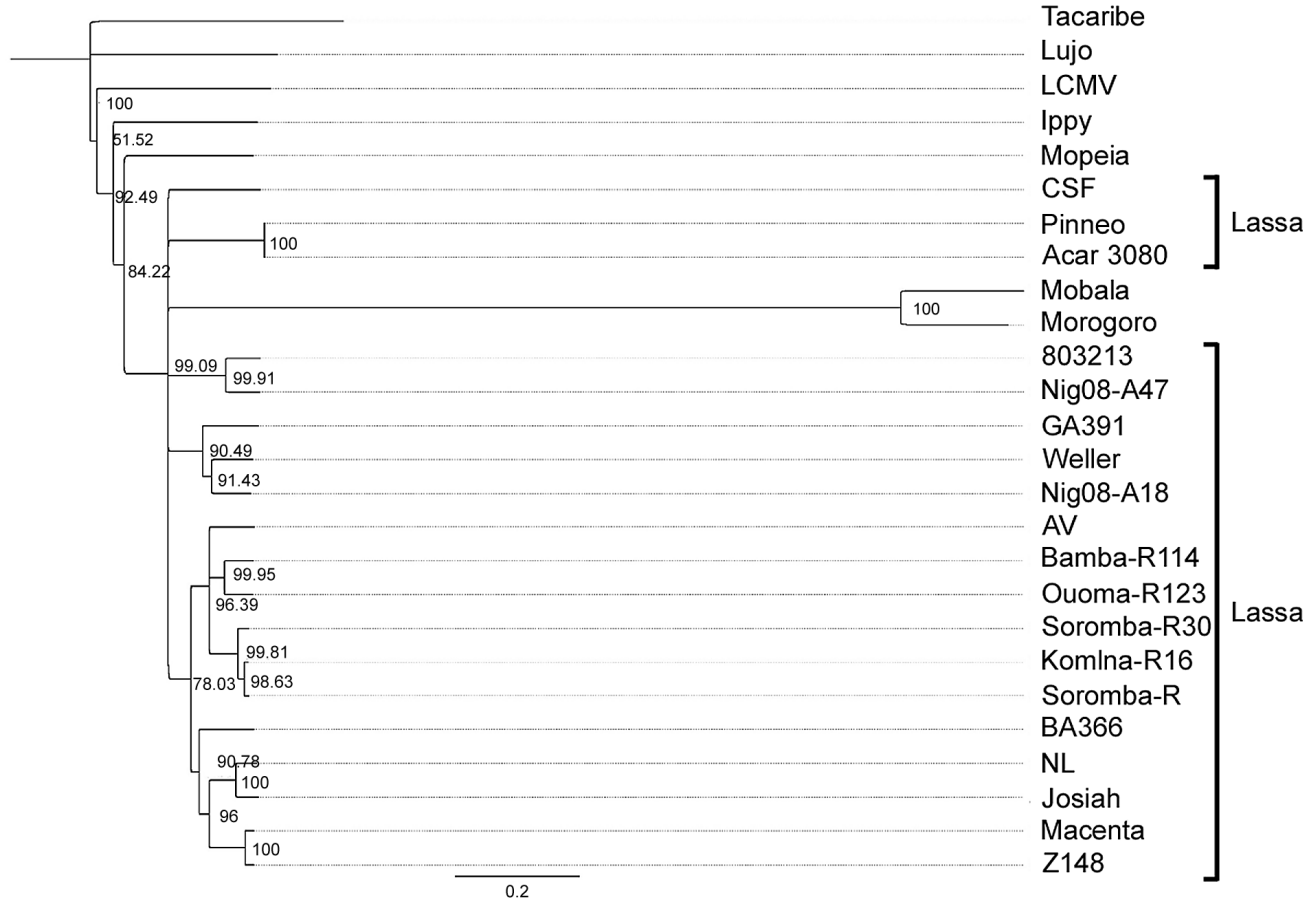
Phylogenetic analysis was conducted on full-length sequences for the nucleocapsid protein using the Jukes-Cantor Neighbor-joining method with 10,000 bootstrap replicates. Sequences from the five Malian isolates were compared to the following arenavirus sequences: Tacaribe (NC_004293), Lujo (NC_012776), Lymphocytic choriomeningitis virus (LCMV, strain Armstrong, AY847350), Ippy (NC_007905), Mobala (AY342390), Morogoro (NC_013057), Mopeia (NC_006575) and Lassa virus strains Josiah (AY628203), CSF (AF333969), NL (AY179173), AV (AF246121), Z148 (AY628205), Macenta (AY628201), BA366 (GU830839), Nig08-A18 (GU481070), Nig08-A47 (GU481078), 803213 (AF181854), Pinneo (AY628207), GA391 (X52400), Acar 3080 (AY628208) and Weller (AY628206).

**Figure 3:**
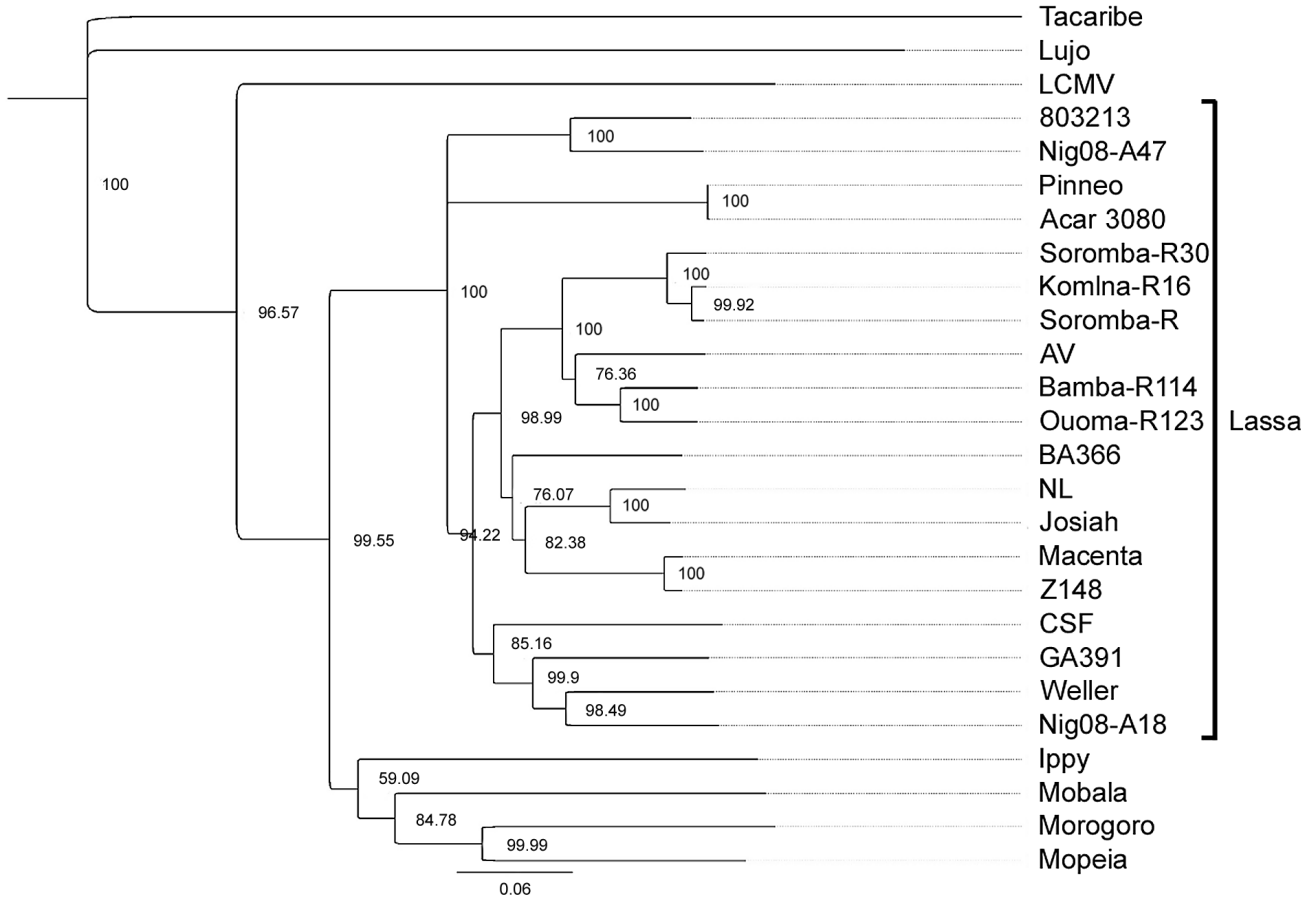
Phylogenetic analysis was conducted on full-length sequences for the glycoproteins using the Jukes-Cantor Neighbor-joining method with 10,000 bootstrap replicates. Sequences from the five Malian isolates were compared to the arenavirus sequences outlined in figure legend 2.

**Figure 4:**
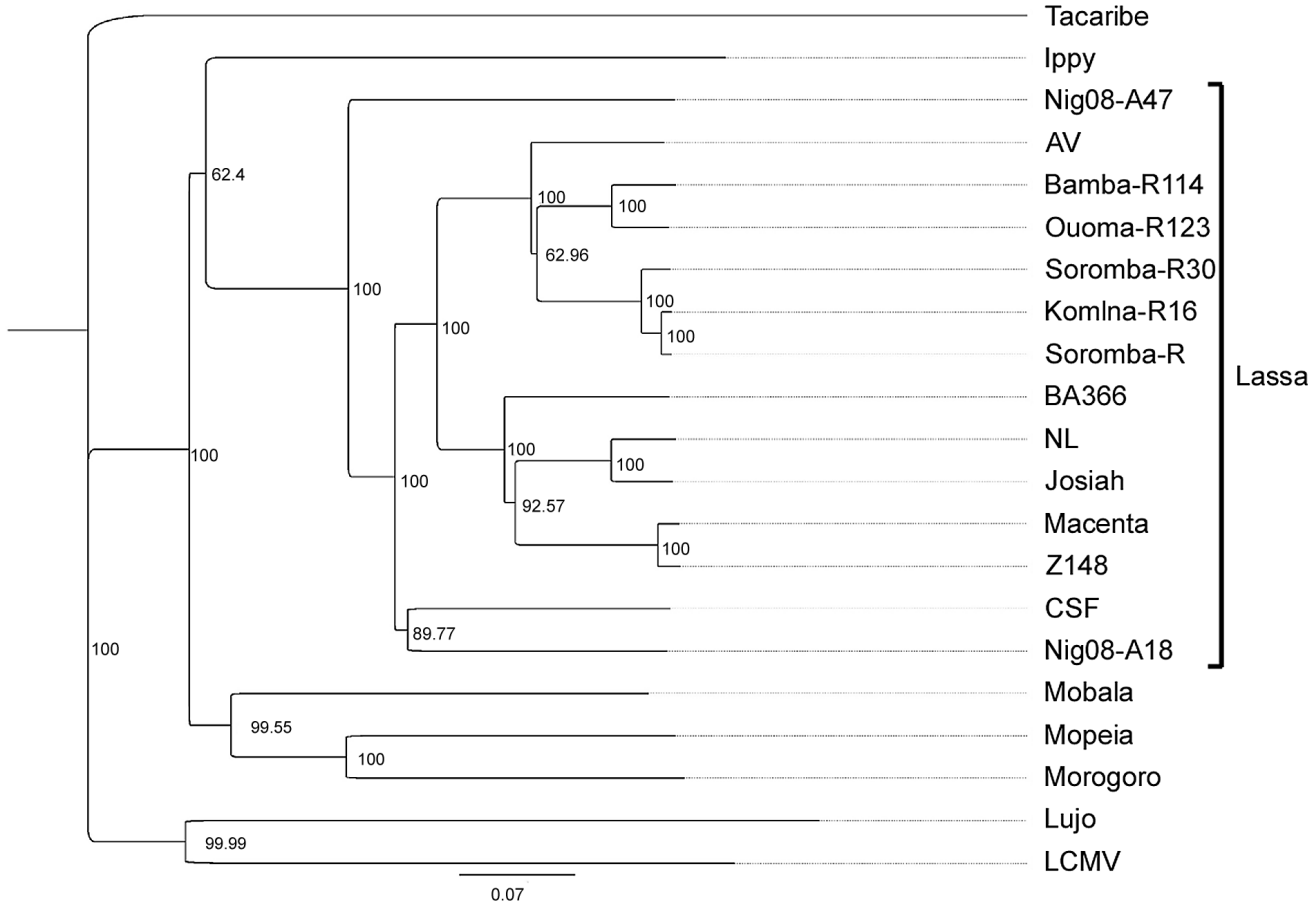
Phylogenetic analysis was conducted on full-length sequences for the polymerase using the Jukes-Cantor Neighbor-joining method with 10,000 bootstrap replicates. Sequences from the five Malian isolates were compared to the following arenavirus sequences: Tacaribe (NC_004292), Lujo (NC_012777), Lymphocytic choriomeningitis virus (LCMV, strain Armstrong, J04331), Ippy (NC_007906), Mobala (NC_007904), Morogoro (NC_013058), Mopeia (NC_006574) and Lassa virus strains Josiah (NC_004297), CSF (AY179174), NL (AY179172), AV (AY179171), Z148 (AY628204), Macenta (AY628200), BA366 (GU979513), Nig08-A18 (GU481071), Nig08-A47 (GU481079).

The full-length genomic sequences of five LASV isolates were determined using next generation sequencing technologies (accession numbers KF478760-KF478769). In addition to the original isolate (Soromba-R) [17], four new LASV isolates were obtained from tissues of rodents captured in March 2012 from the villages of Soromba, Komina, Bamba and Ouoma (designated Soromba-R30, Komina-R16, Bamba-R114 and Ouoma-R123, respectively). Phylogenetic trees constructed with the nucleotide sequences of the nucleocapsid protein (Figure 2), glycoproteins (Figure 3) and polymerase (Figure 4) all demonstrated similar topologies. The Malian isolates grouped closely with strain AV within the LASV lineage and were most divergent from isolates originating from Nigeria. The nucleocapsid gene sequence of the prototype Malian LASV strain Soromba-R differed from AV by 14% at the nucleotide level and 4.2% at the amino acid level. The glycoprotein gene sequences differed by 15.4% at nucleotide level and 4.4% at the amino acid level.

## Discussion

Although Lassa fever was initially described in 1969 and the association with rodents made in the early 1970's, few studies have addressed the geographical distribution of infected rodents beyond Nigeria, Sierra Leone, and Guinea [3], [5], [7]–[9]. Currently, prevention and control of LASV in West Africa relies heavily on educational campaigns aimed at rodent control and avoidance as well as appropriate management of confirmed cases. Field studies like the one described here are important for defining the geographic distribution of LASV infected rodents and can help focus public health preparedness. The results of these studies suggest the risk of exposure to LASV is greatest in the wooded savannas of southern Mali where infected rodents were documented in several villages. The overwhelming majority (>99%) of rodents captured in this area were genetically identified as *M. natalensis*. The lack of other rodents in these villages may support the endemic nature of LASV, since low biodiversity tends to increase pathogen transmission and subsequently the incidence of human disease [26]. It should also be noted that limited field samples were collected in western Mali boarding Senegal and Guinea and therefore the prevalence of LASV infected rodents in this region remains unknown. Ecologically, this area shares many similarities with southern Mali, as well as other West African countries where LASV is considered endemic and therefore the presence of LASV in indigenous rodents should not be ruled out [19].

In light of the findings presented here, appropriate diagnostic tests should be established in southern Mali to help diagnose acute infections and screen individuals in order to better define the burden of infection and disease associated with LASV. Despite an overall prevalence rate of nearly 20% in *M. natalensis*, at present only one confirmed human case of Lassa fever has been documented in Mali. In this study, all rodents with evidence of LASV infection were captured in peridomestic settings, most frequently in kitchens and bedrooms of human dwellings. With these conditions, it seems likely that people are being exposed to LASV. Interestingly, recent studies in the macaque model of Lassa fever suggest the Malian isolates of LASV present with increased pulmonary manifestations, which could be diagnostically misleading especially for local physicians who are not familiar with the wide range of clinical presentations associated with this disease [27]. Combined, the results of our field work and disease modeling efforts highlight the importance of improving the diagnostic capabilities in Mali.

Little is known about the infection dynamics of LASV in the natural rodent reservoir. A limitation to understanding the ecology of LASV infections in wild rodents is the minimal testing conducted with most studies performing either RT-PCR or serology. In our study we employed both methods and the results suggest three distinct patterns of LASV infection in *M. natalensis*; RT-PCR positive, serologically negative; RT-PCR positive, serologically positive; and RT-PCR negative, serologically positive. The first group (RT-PCR positive, serologically negative) most likely represents recently infected animals in which the virus is actively replicating but the host has not yet mounted a measurable humoral response. Alternatively, these animals may represent a group that was exposed to LASV *in utero* (i.e., vertical transmission) and therefore may be unable to mount an effective immune response against the virus. Although the current study samples were not collected to assess transmissibility of LASV from infected rodents, we anticipate this group of rodents represents the greatest threat to human exposure due to unchecked viral replication which presumably would lead to increased shedding. The second group of animals (RT-PCR positive, serologically positive) most likely also represents a threat to humans, though with mounting immune response, these animals may be trending towards viral clearance and therefore might shed less infectious virus. Supporting the hypothesis of viral clearance, Ct values from blood and tissue samples from this group of animals were higher than those in the first group (data not shown), suggesting a lower viral burden. The third group of animals (RT-PCR negative, serologically positive) likely represents those with evidence of a past LASV infection. While serological positivity could denote carry-over maternal antibodies, the animals in this group were all adult which suggests maternal antibodies had waned and the detection of anti-LASV antibodies was due to previous infection. These animals are not likely to represent a threat to human exposure, though we cannot exclude the possibility of the presence of LASV in tissues other than those tested here, which could reactivate and result in an active infection and subsequent transmission of virus.

The predilection for *M. natalensis* to inhabit peridomestic settings inhibits mark-recapture studies to better define the ecology of LASV infection in these animals. For example, it would be ethically unacceptable to release a known LASV-infected rodent back into a human dwelling. Therefore, the only way to elucidate LASV infection dynamics in individual rodents is through experimental infections in a controlled laboratory setting. To date, only one study has addressed experimental infections in *Mastomys natalensis*; however the emphasis was on pathology and not infection kinetics, host responses and most importantly transmissibility of LASV [28]. Similar to previous work with hantaviruses, efforts should be made to model the rodent host/pathogen interactions for LASV in a laboratory setting to decipher the patterns of infection [29].
